# Gender and intersectional barriers to child immunization: findings from qualitative research in Nigeria

**DOI:** 10.1186/s12939-026-02802-5

**Published:** 2026-02-28

**Authors:** Maria Klara Kuss, Lily Breimann, Kiran Stallone, Alinane Kamlongera, Ibrahim Dadari, Miguel A. Rivera-Quiñones

**Affiliations:** 1 University of Bonn-Rhein-Sieg, Sankt Augustin, Germany; 2 Ladysmith Collective, British Columbia, Canada; 3 UNICEF Headquarters, New York, USA; 4 Radboud University, Nijmegen, The Netherlands

**Keywords:** Child vaccination, Care work, Gender, Motherhood, Intersectionality, Nigeria

## Abstract

**Background:**

Preventable diseases remain a leading cause of child mortality globally, with routine vaccination critical to saving lives. Research has increasingly examined the reasons behind low child immunization. While gender is recognized as important, most studies treat women caregivers as a homogeneous group, overlooking how intersecting factors like age, religiosity, and marital status shape vaccine access. This study fills this gap by analyzing how different social categories interact with gender to influence caregivers’ experiences with child vaccination in Nigeria.

**Method:**

A novel conceptual approach was used to capture gender-related and intersectional barriers to vaccination across both supply and demand sides, spanning multiple socio-ecological levels. The study employed a qualitative research design, including a desk review, 67 key informant interviews, and 36 focus group discussions. Primary data were collected across six Nigerian states: Lagos, Kaduna, Cross River, Gombe, Ebonyi, and Kwara. Participants were purposively selected to reflect diverse caregiving experiences. Thematic analysis guided interpretation, with findings compared across caregiver groups through an intersectional lens.

**Results:**

The article shows that different groups of women caregivers – married women, devout women, adolescent mothers, and grandmothers – face different challenges when seeking to vaccinate their children in Nigeria. Married women must negotiate the benefits and costs of vaccination with their husbands. Devout mothers have to balance scientific knowledge with deeply held religious beliefs. Adolescent mothers juggle their limited status as young women, lack of knowledge, and negative experiences with health workers in the process of seeking vaccination for their children. Meanwhile, grandmothers manage to promote vaccination and provide hands-on support but face challenges like overcrowded transport and long waits.

**Conclusions:**

The article concludes that gender-just child immunization requires a care-integral approach that recognizes the inequities different groups of women caregivers experience in accessing and using child vaccination services. However, simply acknowledging their distinct challenges is not enough. Gender-just programming must recognize the critical role and value of women caregivers in child vaccination and ensure a more equitable distribution of resources to support them.

## Background

Globally, an estimated 14.5 million children failed to receive any vaccines at all in 2023, with an additional 6.5 million not completing their vaccination schedule (UNICEF, [35]). Nigeria, specifically, faces persistent challenges as vaccination coverage remains low nationally, with over 2 million zero-dose children – representing nearly 14% of the global total (GAVI, 2021; UNICEF, [34]). Alarmingly, only 36% of children aged 12–23 months have received all recommended immunizations, while 18% received none (MICS and NICS, [26]).

Scholarly and policy research has paid growing attention to the reasons and determinants of low child immunization coverage and inequity^1^ [1, 22, 28]. A key stream within this work focuses on the role of gender in influencing vaccine access, uptake, and coverage globally [11, 21, 33]. Existing studies suggest that gender-related barriers may be among the most widespread factors impeding equitable vaccine coverage in low- and middle-income countries [2, 21, 30]. This is largely because women are typically responsible for children’s health and vaccinations. They must bring children to health centers, follow up on appointments, obtain accurate information, and persuade family or community members—including husbands—of the benefits of vaccination. Yet, they often lack the resources, autonomy, knowledge, or authority needed to carry out and navigate these responsibilities [21, 25].

While offering valuable insights, much of the literature on women’s roles in child vaccination treats women as a homogeneous group, often overlooking the diverse and intersecting realities of women’s lives, including differences in class, education, ethnicity, marital status, and geographic location. Instead, women or mothers are often generalized as a singular, homogenous category. This categorization is problematic not only as an academic oversight but also because research into the gender barriers of child vaccination can shape and inform real-world immunization programming. Without recognizing the layered and varied experiences and challenges of different women caregivers, interventions may inadvertently reinforce existing power imbalances and overlook the specific challenges and struggles of different caregivers in accessing child immunization.

Recognizing this oversight, there have been growing calls for a more nuanced, gender-intersectional approach to child immunization research [11, 21]. Intersectionality provides a framework to examine how different social categories—such as gender, race, class, and age—interact to shape immunization and other outcomes (see [24]). Historically, studies of intersectionality have often focused on interactions between gender and race as systems of power, particularly on how these two come together to compound discrimination. Foundational theorist and activist Kimberlé Crenshaw famously argued that we create further marginalization by focusing solely on gender or race “as mutually exclusive categories of analysis” ([8], p. 139). She underscored that, by doing so, we miss the “intersecting patterns of racism and sexism” ([9], p. 1244). By thinking about gender and race *as categories of analysis* as opposed to *identities of analysis*, Crenshaw emphasized the systemic nature of marginalization—that gender and race operate as socially constructed systems of power rather than inherent characteristics. As such, intersectionality offers “an analytic framework that expose[s] […] exactly what it mean[s] for systems of power to be interactive” ([7], p. 3).

Scholars are beginning to expand traditional notions of intersectionality to consider how other societal structures and categories interact to impact child immunization access and uptake [5, 19, 30, 31]. As Cha et al., [5] highlight, the likelihood of an individual receiving recommended vaccines is shaped by a range of determinants - such as gender, socio-economic status, geographic location, and others. These factors often overlap, compounding upon one another to create barriers or opportunities for vaccination. Cha et al. [5] therefore advocate for incorporating an intersectional lens in immunization research to address health inequities and increase vaccine coverage. Scholarship on child immunization also strongly advocates for using an intersectional lens to understand the multifaceted barriers faced by caregivers. For instance, Feletto and Sharkey, [11] emphasize that the caregiver experience is shaped by a variety of social, institutional, and structural factors that can either empower or disempower them to vaccinate their children.

Despite calls for further research, studies that explicitly apply an intersectional approach to child immunization remain limited, particularly in the African context. A notable exception is Shearer et al.,’s [30] study on the drivers of childhood immunization inequality in the Democratic Republic of Congo, Mozambique, and Nigeria. This study applies an intersectional approach and a human-centered design to gain insights into the different experiences of caregivers in their efforts to vaccinate their children. It highlights how socio-economic status (wealth) and geographic location (urban/rural, access) intersect with gender to create barriers for caregivers. However, the study sidelines other intersecting categories, such as marital status, religiosity, and age. Our research builds on this existing work by studying the role of these particular factors in shaping caregiving responsibilities for child vaccination in Nigeria.

## Methods

This study examines how gender and intersectional factors influence caregivers’ experiences with child vaccination in Nigeria. Specifically, it investigates the following factors: marital status, religiosity, and age. To operationalize our research question, a novel conceptual approach based on a comprehensive review of gender, health, and vaccination literature was developed. This framework was later published as UNICEF’s Global Gender Analysis Tool for Gender and Immunization (UNICEF, [36]). The framework accounts for gender-related and intersectional barriers to vaccination on both the supply and demand sides, across individual, household, community, health facility, and policy levels. Importantly, it allows for an analysis of the varying and intersectional categories that shape how different groups and individuals access and use vaccination services.

Methodologically, this study used a mixed-methods design comprising a desk review, Key Informant Interviews (KIIs), and Focus Groups Discussions (FGDs). The process began with a desk review to establish a foundational understanding of existing evidence and identify key knowledge gaps. This review informed the subsequent primary qualitative data collection, which aimed to explore the direct experiences and views of policy managers, implementers, healthcare workers (HCWs), religious and community leaders, as well as mothers and fathers, with child immunization. Data collection consisted of 67 KIIs to capture expert and institutional perspectives, and 36 FGDs to gain in-depth insights into caregivers’ unique experiences shaped by different social categories, the health system, and the broader structural context.

Primary data collection was undertaken in six states – Lagos (South-West), Kaduna (North-West), Cross River (South-East), Gombe (North-East), Ebonyi (South-East), and Kwara (North-Central). The aim of the state selection was to capture a comprehensive picture of Nigeria’s main geopolitical zones, and thus to obtain a sample representative of Nigeria. The selection of states was based on the following predefined criteria: lowest coverage of the third dose of the pentavalent vaccine, notable gender gaps, security situation, and diverse vaccine performance. For comprehensive representation, the fieldwork sites in each state were carefully chosen to include both urban and rural settings.

Participants were purposefully selected to ensure the inclusion of diverse groups with varying experiences in relation to child vaccination. Specifically, the sampling was designed to capture multi-level perspectives on gender and intersectional barriers, from policy formulation to frontline service delivery, community-level decision-making, and the realities of individuals on the ground. Accordingly, KIIs were conducted with representatives of international organizations, the Ministry of Health, the Ministry of Women Affairs (national level), representatives of the National Primary Health Care Development Agency (NPHCDA) and State Primary Health Care Development Agency (SPHCDA) (subnational level), and religious leaders, community leaders, and healthcare workers (community level). To reach relevant interviewees, the research team leveraged the networks of UNICEF country office and the Ministry. FGDs at the community level were conducted with fathers, mothers, and health care workers (with an average of eight participants per group). FGDs with mothers and fathers included a diverse range of participants, including married mothers, adolescent mothers, grandmothers, women from female-headed and polygamous households, mothers of both younger and older children, and male spouses. To reach potential participants, the research team worked with local contacts to introduce the study to community members, confirm their participation availability, and schedule dates for the FGDs. See Table 1 for an overview.

Data collection was conducted from September to December 2023 by a team of national researchers and translators who were well acquainted with the local context and research sites. Prior data collection, ethical clearance was obtained from the National Health Research Ethics Committee (NHREC/01/01/2007-08/09/2023B), and the data collection team received training on the research objectives, ethical procedures, data collection methods, and research instruments. The team also piloted and practiced the instruments to ensure they could elicit in-depth responses through ethical and effective probing, and to confirm that the questions were culturally appropriate and clearly understood. To access participants and communities, the team held phone consultations with key stakeholders (e.g., National and State Primary Health Care Development Agencies, and community leaders) to introduce the study, identify informants, and schedule FGDs and KIIs. Scheduling the FGDs and KIIs, in particular, proved time-consuming and contributed to delays in the study timeline.

All participants provided informed consent prior to their involvement and were informed of their right to withdraw at any time without penalty, including giving permission to audio record the interviews. All KIIs and FGDs were audio recorded, then transcribed and translated into English for analysis. All data was anonymized, and personal identifiers were removed and stored securely to ensure confidentiality.

Although we developed interview questions that related to the different intersectional categories noted above, in the analysis phase, we were open to the wide range of experiences and insights that emerged about those categories. This approach aligns with an Interpretive Description (Thorne [32]). Indeed, Thorne ([32], 18) writes that this approach:

*constitutes a methodological direction that generates questions from applied disciplinary grounding, pushes one into the “field” in a logical, systematic, and defensible manner, and creates the context in which engagement with the data extends the interpretive mind beyond the self‑evident—including both accumulated knowledge (such as clinical wisdom) and what has already been established (such as through empirical means)—to see what else might be there.*

In line with the above, this article pooled the data to focus on comparisons across different socially constructed categories and focused on the unique insights that emerged about them from participants. As such, the analysis reflects the experiences of our participants – such as married mothers, adolescent mothers, devout mothers, and grandmothers – and captures diverse perspectives on child immunization access and barriers. In the next section, the results are presented according to these participant groups. Quotes from the KIIs and FGDs are referenced using identifier codes that specify the data type (KII/FGD), socio-ecological level, state, specific site, and respondent type.

## Results: gender- and intersectional barriers to child vaccination

Although the NPHCDA and SPHCDA provide basic immunizations free of charge, equitable access remains a challenge. This section explores how different groups of caretakers encounter uniquely different barriers when seeking to vaccinate their children. It illustrates this by highlighting key challenges faced by married mothers, adolescent mothers, devout mothers, and grandmothers.

### Married mothers

Married mothers face a complex array of barriers when it comes to accessing immunization services for their children. Our findings suggest that in married couples’ households in Nigeria, it tends to be the mother who takes on the sole responsibility of childcare, including immunization, with limited paternal involvement. This dynamic is rooted in societal norms that define a wife’s primary role as caring for the home and children, while the husband holds authority over both public and private matters. For example, Nigeria’s MICS & NICS survey found that only 10% of fathers of children aged 24 to 59 months engaged in four or more responsive care activities in Nigeria (MICS & NICS, [26]). While our KIIs and FGDs suggest that some men may be more involved in caregiving, they show that social norms and community attitudes often discourage male participation in caregiving tasks. This study found that, in some areas, men may even face social stigma when willing to support care work associated with child vaccinations: “[If] you see a man bringing a child for vaccination like in our typical Nigerian context, most people would be wondering like ‘where is the mother, that the father is the one bringing the child for immunization?’” (KII-N-PO-2).

The research findings also show that patriarchal decision-making further complicates uptake of immunization services by married women as it restricts their autonomy. Despite being primary caregivers, they often lack the authority to make healthcare decisions for their children, as social norms place this responsibility with their husbands. In many communities across Nigeria, husbands control key healthcare decisions, including those about their children’s immunization. These dynamics require married mothers to seek their husbands’ permission before accessing immunization services for their children. Many women face resistance from their husbands, who may be sceptical of vaccination or outright forbid it, leaving mothers in a difficult position where they must either comply or attempt to persuade their spouses. Our findings show that in some cases, women try to hide their children’s vaccinations from their husbands to avoid conflict, while others emphasize the importance of convincing men of immunization’s benefits. This finding is supported by Antai’s [2, p. 136) study, which found that Nigerian “children of women who lacked decision-making autonomy had a lower likelihood of receiving full childhood immunization.” As illustrated by one of our female national key informants from Kaduna:

*For a woman*,* if your husband does not give you permission*,* no matter how knowledgeable you are about immunization*,* you cannot take your baby to the hospital for vaccination or any vaccination center. You cannot even give your baby [vaccines] even if it is during [vaccination] campaigns because you are afraid of what might happen (KII-SN-K-N).*

While this finding applies across Nigeria, it is more pronounced in Northern Nigeria than in the South, where married women’s autonomy is more constrained in the more conservative social context. As a religious leader in Gombe stated, “This is Gombe, not Lagos. Here, men have superiority so they are the ones steering the affairs of the house” (KII-C-G-S-RL).

Married mothers’ financial insecurity and economic dependence on their husbands poses another important barrier to accessing immunization services. Even when they engage in income-generating activities, married mothers are often confined to informal, low-paying jobs with little job security or financial independence. Additionally, in many cases, men are expected to be the primary financial providers, which reinforces married mothers’ reliance on their husbands for basic needs, including healthcare expenses. Hence, married mothers may be unable to stem costs associated with transportation or other informal fees related to immunization services – such as payments for registration cards, weighing children, or even paying the guards at immunization centers. A subnational respondent explained that “[if] women are not empowered, they are not economically empowered, they don’t have a source of livelihood and depend on husbands. So […] the mother said her children cannot be vaccinated” (KII-SN-G-S).

Another key barrier to child vaccination among married mothers is the widespread fear that vaccines, particularly the polio vaccine, may cause infertility. This belief tends to be more prevalent in northern states, where vaccine hesitancy is sometimes linked to narratives of Western interference and shaped by broader historical mistrust in government institutions and health systems [20]. Interviewees from Kaduna, Gombe, and Kwara highlighted that rumors targeting women’s fertility contribute to this fear: “Some are afraid of polio vaccines because of the rumors. […] They say the vaccination is not against polio but against fertility” (KII-C-G-S-RL). This misinformation creates anxiety among married mothers, who may avoid vaccinating their children—especially their unmarried daughters—out of concern for their future fertility and marriage prospects. For women whose value is closely tied to childbearing, the social risks of being seen as infertile are significant. These fears also influence male spouses, who often control health-related decisions, further hindering vaccination.

### Adolescent mothers

In Nigeria, around one in five mothers (19%) were between 15 and 19 years old in 2018 (Bolarinwa et al., 2023), highlighting adolescent mothers as an important target group for child immunization programming. Our findings show that intersecting factors—age, gender, and social status and norms—limit adolescent mothers’ ability to access immunization services for their children. In particular, the widespread requirement for parental or male permission undermines their autonomy in making even healthcare decisions, including child vaccination. As such the decision-making constraints outlined in the previous section are intensified for adolescent mothers, who often defer to male authority—whether fathers, husbands, or community leaders—before vaccinating their children. Healthcare workers, influenced by such social norms, sometimes inadvertently reinforce the expectation that adolescent girls need male or parental approval to vaccinate their children. As one key informant explained,

*An adolescent girl [is] not able to do anything without the permission of her parents*,* she can’t just walk into the clinic and say ‘give me [or her child] vaccination’ – even the healthcare workers by reflex would ask her of her mother (KII-SN-G-M-2).*

Moreover, adolescent mothers often lack financial independence. Their young age and responsibilities as mothers limit their ability to secure stable jobs or reliable sources of income, creating challenges in covering transportation costs or informal fees associated with healthcare services. Thus, adolescent mothers are heavily reliant on their fathers or husbands for financial support. As reported by an adolescent mother: “[My husband] assists in making provision for transport to drop meat the immunization place and also pick me up; and sometimes gives me additional money in case of any emergencies” (FGD-C-G-S-M).

While our data do not allow us to fully explore how age and marital status interact to shape these experiences, the findings highlight how social stigma from healthcare workers hinders adolescent mothers from vaccinating their children. Respondents described judgmental attitudes from healthcare workers that create an unwelcoming environment, leaving adolescent mothers feeling ashamed or embarrassed when seeking services. These attitudes discourage adolescent mothers from returning for vaccinations, ultimately lowering immunization uptake. As one adolescent mother from Gombe shared, “The health workers look down on me because I’m an adolescent mother” (FGD-C-G-G-M). In addition, respondents indicated that some healthcare workers subject adolescent mothers to verbal humiliation, further discouraging them from visiting health facilities. For example, one participant described how adolescent mothers are sometimes ridiculed, saying, “Some, [HCWs] give them embarrassment. […] some nurses will go and abuse her ‘See how small you are’. If a girl comes once or twice [to the clinic] and that embarrassment happens, because of that embarrassment it makes her stop going” (FGD-C-CR-A-M).

Further, our findings indicate that adolescent mothers, particularly those in early marriages, often lack adequate information about their healthcare rights, including immunization. As one key informant explained, “Do they [adolescent mothers] even know that the adolescent can access family planning? -They don’t know, so they need somebody who will encourage them, teach them. […] So, it is a behavioral change, and you need to create that awareness in the mind of the people” (KII-SN-E-SPHCDA). This gap in knowledge is especially concerning, given that approximately 30% of women aged 20 to 24 were married or in union before the age of 18 (MICS & NICS, [26]).

### Devout mothers

For devout mothers, particularly in communities with strong Christian or Muslim beliefs, several barriers hinder their uptake of immunization services for their children. Our findings indicate that one important barrier is the influence of religious doctrines that affect mothers’ willingness to vaccinate their children. For example, in some Nigerian religious communities, devout mothers are taught to rely solely on faith for protection, leading them to reject drugs and vaccines. As illustrated by an interviewed health worker, “I have experiences where you [Health Care Workers] go to church […] and they [mothers] will be like they don’t need it [vaccinations]. They don’t believe in it. Sometimes they rejected it outrightly […] they deliberately rejected it” (KII-C-CR-CM-HCW-1). This finding aligns with Anyene (2014, p. 7), who notes that “in some Christian communities, there are a few sects that hold to the doctrine of non-use of drugs, relying only on one’s faith in all circumstances. This extends to immunization of children.”

Misinformation within religious communities further compounds these challenges. Devout mothers are particularly vulnerable to false claims—such as vaccines causing infertility, death, or birth defects—that circulate widely in religious and ethnically homogenous areas, especially in Northern Nigeria (Nasiru et al., 2012; Mahachi et al., 2022). A key informant recalled that a mother believed her “cousin had an encounter at a health center when taken for immunization and she was injected wrongly. This made her deaf and dumb. This has discouraged a lot of mothers from taken their children to health center due to incompetency. It is better to pray before going” (KII-C-L-HF). In many cases, trusted religious leaders reinforce these beliefs, shaping how devout mothers perceive immunization. As one leader explained, “When that [vaccination] strategy comes to us, we look at it first—how does the Islamic perspective look at that strategy […] if the advantages outweigh the disadvantages, provided it does not affect your religion, it will be allowed” (KII-C-CR-CM-RL). Such messages create confusion and distrust, making it harder for devout mothers to align their faith with vaccination advice.

Devout mothers also face structural access barriers due to gender norms rooted in religious practices. For example, our findings, particularly from Northern states, indicate a strong preference for female healthcare workers in Muslim communities, where it is often prohibited for men outside the household to interact with female family members. As one health worker from Gombe explained, “In some places, particularly in the Muslim setup, they prefer women to get into their houses to vaccinate” (KII-C-G-S-HCW-2). A subnational informant from Gombe added, “The truth is that entering households in Northern Nigeria is synonymous with using female healthcare workers. For you to get mileage on immunization especially when it comes to entering households in Northern Nigeria, it’s best to use female healthcare workers.” (KII-SN-G-M-2). These norms limit devout mothers’ access to vaccines and highlight the need for gender-sensitive delivery strategies, including the deployment of female health workers to reach mothers in their homes.

### Grandmothers

Grandmothers, particularly in multigenerational households, often play a pivotal role in caregiving and healthcare of their grandchildren in Nigeria. However, their involvement can present specific challenges to child vaccination. Our findings suggest that grandmothers play an important advisory role in decisions about child healthcare. When supportive of vaccination, they can encourage timely immunization and remind mothers about upcoming appointments. However, their influence can also act as a barrier: skepticism toward vaccines, rooted in preferences for traditional remedies such as palm wine or herbal concoctions, leads some grandmothers to discourage vaccination. As one respondent noted, “In some areas, they [grandmothers] believe that once they take their herbal concoction, they are covered and don’t need anything else” (KII-SN-CR-SPHCDA). To add to that, some older generations view vaccines as Western or foreign interventions that conflict with traditional practices. A representative from the Ministry of Women Affairs in Gombe shared, “It is a norm, especially among older generations, to see vaccines as a Western thing that is brought to us, so we don’t accept it at once” (KII-SN-G-M-1). For these grandmothers, vaccinating a child may feel like rejecting locally accepted healing methods. These beliefs were especially prominent in rural areas, which are characterized with higher illiteracy rates, and less access to information and education. In such contexts, the views of grandmothers can place mothers in a difficult position, as choosing to vaccinate their children may be perceived as challenging the authority and wisdom of respected older family members.

We also found that grandmothers can provide critical hands-on support by taking grandchildren to health facilities, thus easing the burden on mothers. Yet, their ability to do so is often constrained by structural barriers. Poor infrastructure—including long distances, limited transport, inadequate facilities, and long wait times—disproportionately affects older women with mobility or stamina limitations. For example, grandmothers experience physical limitations that make it difficult to travel long distances to vaccination centers, a problem that is especially acute in rural areas where services are sparse and transport options are few. One grandmother from Cross River articulated this challenge and proposed a solution: “The hospital should be brought close to us so that it will be easy for us grandmas” (FGD-C-E-I-M). Moreover, the findings indicated that many healthcare facilities lack basic amenities such as sanitation, seating, and reliable power supply, making it difficult for older women to endure long waits or uncomfortable conditions. A representative from a partner organization explained,

*We noticed a sudden decline in immunization uptake and we tried to find what happened. The [elderly] women had only one issue*,* which was that they got to the facility and there was no [sanitary facility] to ease themselves. That caused a drastic decline in uptake. In another place*,* they have nothing to sit on*,* no bench (KII-N-PO-3).*

## Discussion: towards a care-integrated approach to child immunizations

Child vaccination involves care work – work that is unpaid, time-consuming, difficult and typically performed by women. To get children vaccinated, women must organize their time and labour while navigating complex social relationships to overcome a range of challenges and barriers. Existing scholarship on gender and child vaccination often highlights gender-related demand and supply-side barriers such as control over resources, competing responsibilities, education, availability of female health workers, long distances to vaccination centers, and lack of basic infrastructure at vaccination points. While these are important factors and considerations, this work rarely examines the more specific ways in which gender intersects with other social categories to influence child immunization. Instead, women and/or mothers are often generalized as a singular, homogenous category. There is a broad assumption that their experiences are the same simply because they are *women* and simply because they are *mothers*. Additional interactions between gender and other social categories, like age, religiosity and marital status, tend to be overlooked.

By highlighting the perspectives of different women caregivers involved in child vaccinations in Nigeria, this article has offered an alternative approach to understanding women’s experiences. Across most research sites, married women need to get permission from their husbands to vaccinate their children. They must also overcome fears such as those surrounding infertility and marriage prospects of their daughters, and figure out how to get money for transport and informal fees in a context where the limited household income is already needed for basic needs like food. Adolescent mothers also often struggle to navigate negotiations with their husbands or parents. As young women, they may lack the status within their households that adult women have, and their limited knowledge about the importance and benefits of vaccination further hinders their ability to effectively communicate and negotiate with family members. They might also be discouraged to go to the vaccination appointments if they feel disrespected by health care workers.

Meanwhile, devout mothers navigate the complexities of balancing scientific knowledge with deeply rooted religious beliefs within their communities. This balancing act can involve resistance from religious leaders and others who question the safety or legitimacy of vaccination, particularly when it conflicts with traditional teachings. For these women, the decision to vaccinate is not only a matter of household decision-making but involves a balance with religious and community norms. Lastly, grandmothers can either support or hinder vaccination efforts, depending on their stance toward modern healthcare practices. If supportive, they may provide hands-on help by taking their grandchildren to health facilities, thus easing the burden on mothers. However, this help comes with its own challenges – including exhausting and overcrowded transportation on difficult roads or long waiting times in uncomfortable waiting areas.

*Recognizing* the needs and challenges faced by different groups of women is a crucial step toward making their care work - and their experiences and difficulties with child vaccination - visible: their struggles persuading hesitant husbands, navigating religious and community norms around child vaccinations, their time sacrifices and their physical and emotional labor. Practice-oriented scholarship (e.g. Gerber et al., [17], Zasada, [37]) that is geared towards increasing child vaccination rates, typically emphasizes that design and implementation of interventions should be adjusted to reduce these gender-related barriers. Programmatic recommendations may include improving transportation and infrastructure to support grandmothers willing to assist with immunization efforts, launching social and behavioral change initiatives aimed at encouraging fathers to permit their wives to take children to vaccination appointments, or training health care workers in gender-sensitive and adolescent friendly service delivery. Several of these are being prioritized in some countries as pro-equity immunization interventions [10].

However, such solutions pose a *dilemma*. While they may recognize the unpaid care work and the challenges of different groups of marginalized women, they tend to adopt an instrumental logic—treating women’s labor primarily as a means to improve child health outcomes. In doing so, they risk reinforcing traditional gendered divisions of labor by placing the responsibility of children’s health squarely on women, rather than challenging the norms and expectations around women’s roles and caregiving. These critiques are well-established in feminist scholarship on reproductive and productive labor [3, 12, 18, 29]. Yet, at the same time, it is clear that, in practice, women continue to bear the day-to-day responsibility for caregiving—an arrangement deeply embedded in prevailing social structures and norms that are resistant to change. This arrangement creates a tension: while we may be critical of approaches that entrench unequal responsibilities, there is also an ethical imperative to support women in the current realities they face. As Lister [23] puts it:

*We are torn between wanting to validate and support […] the caring work for which women still take the main responsibility in the private sphere and to liberate them from this responsibility so that they can achieve economic and political autonomy in the public sphere. (*[23], p.*178)*

This dilemma between supporting and liberating women’s care work reflects long-standing feminist debates about social justice and emancipatory politics (e.g. Butler, [4], Fraser, [13], Fraser, [15], Fraser, [16]. Notably, it reinvigorates Nancy Fraser’s theory of gender justice [13], which distinguishes between two key societal injustice solutions: recognition and redistribution. *Recognition* involves acknowledging and responding to the needs of marginalized groups. While this type of solution “affirms” existing gender roles, it recognizes and seeks to support unpaid care and domestic work [13]. *Redistribution*, by contrast, looks at moving resources across society to dissolve gender differences in access to resources. This type of solution is seen as being of “transformative” nature as it seeks to overturn institutionalized subordination [13]. Fraser contends that, in order to ensure gender justice, both types of solutions are necessary. As she puts it, “social justice today, in sum, requires both redistribution and recognition; neither alone will suffice” (Fraser, [14], p. 149).

This perspective suggests that achieving gender-just child immunization in Nigeria requires a care-integral approach (see [6]) that recognizes the inequities that different groups of women caregivers experience in accessing and using child vaccination services, and implements practical measures to support them. Achieving this demands a clear understanding of the diverse lived experiences, needs, and socio-political realities that shape women’s roles as caregivers — including their ability to vaccinate their children and the time, effort, and costs involved, as illustrated throughout this article. A one-size-fits-all solution — irrespective of age, marital and socio-economic status, religiosity, or geographic location — risks reproducing existing inequities. Moreover, it requires recognizing the value and critical role of women in child vaccination and ensuring a more equitable distribution of resources to support them. This goes beyond micro-level interventions that incentivize both parents to participate equally in children’s healthcare and requires coordinated action at the meso- and macro-levels. Examples include investing in mobile vaccination units and flexible clinic hours to accommodate women’s time constraints, sensitizing community and religious leaders, improving basic infrastructure at vaccination points (e.g., sanitation, seating, and reliable power supply), and training healthcare workers to better support young mothers.

## Conclusions

This article has applied an intersectional lens to understand how different social categories interact with gender to shape child immunization. Drawing on qualitative findings from Nigeria, it revealed how different women caregivers – married women, devout women, adolescent mothers, and grandmothers – navigate child immunization in distinct ways. The findings show that each group faces unique challenges in vaccinating their children. Married women must negotiate the benefits and costs of vaccination with their husbands. Devout mothers have to balance scientific knowledge with deeply held religious beliefs. Adolescent mothers juggle their limited status as young women, lack of knowledge, and negative experiences with health workers in the process of seeking vaccination for their children. Meanwhile, grandmothers, as older women, may manage to promote vaccination and provide hands-on support but face challenges like overcrowded transport and long waits.

Ultimately, the article contends that gender-just child immunization requires a care-integral approach that builds on a nuanced understanding of the diverse circumstances of women caregivers and a recognition of their distinct challenges. However, simply acknowledging these barriers is not enough. Gender-just programming must recognize the critical role and value of women caregivers in child vaccination and ensure a more equitable distribution of resources to support them. To achieve this, future research and policy efforts should explore more deeply the contextual factors that generate inequities among different groups of women and social categories and identify how immunization programming can address them.

**Table 1:** Sampling and research sites

Socio-Ecological Level	Respondent Type	Method	Geopolitical zone							Total
			Abuja	Kaduna	Lagos	Cross Rivers	Ebonyi	Gombe	Kwara	
National	Partner Organizations	KII	5	0	0	0	0	0	0	**5**
	MoH, MoWA, MOFAWA	KII	2	0	0	0	0	0	0	**2**
Subnational	NPHCDA, SPHCDA	KII	0	3	1	1	1	1	1	**8**
	MoH, MoWA, MOFAWA	KII	0	1	0	1	1	2	1	**6**
Community	Religious Leaders	KII	0	2	2	2	2	2	2	**12**
	Community Leaders	KII	0	2	2	2	2	2	2	**12**
	HCWs	KII/FGD	0	4	6	6	6	6	6	**34**
	Fathers	FGD	0	2	2	2	2	2	2	**12**
	Mothers (adolescent, adult, grandmothers)	FGD	0	2	2	2	2	2	2	**12**
**Total**			7	16	15	16	16	17	16	**103**

## Data Availability

The qualitative data, including interview and focus group transcripts, is not publicly available in accordance with Nigeria’s National Health Research Ethics Committee (NHREC) approval.

## Footnotes

1 Drawing on Plamondon and Shahram [27], equity in health is a benchmark for a society where all children can reach their full health potential, without systemic disadvantage. Hence, inequity refers to the unfair and avoidable differences in vaccine coverage that arise from an unjust distribution of power and resources, preventing children from reaching their full health potential.
